# Long-term low-dose tolvaptan efficacy and safety in SIADH

**DOI:** 10.1007/s12020-023-03457-w

**Published:** 2023-07-28

**Authors:** Marta Bondanelli, Ludovica Aliberti, Irene Gagliardi, Maria Rosaria Ambrosio, Maria Chiara Zatelli

**Affiliations:** Section of Endocrinology, Geriatrics and Internal Medicine, Dept of Medical Sciences, University of Ferrara, Ferrara, Italy

**Keywords:** SIADH, Hyponatremia, Tolvaptan, Low-dose

## Abstract

**Purpose:**

Tolvaptan, a selective vasopressin V2-receptor antagonist, is approved for the treatment of SIADH-related hyponatremia, but its use is limited. The starting dose is usually 15 mg/day, but recent clinical experience suggests a lower starting dose (<15 mg/day) to reduce the risk of sodium overcorrection. However, long-term low-dose efficacy and safety has not been explored, so far. Aim of our study is to characterize safety and efficacy of long-term SIADH treatment with low-dose Tolvaptan.

**Methods:**

We retrospectively evaluated 11 patients receiving low-dose Tolvaptan (<15 mg/day) for chronic SIADH due to neurological, idiopathic and neoplastic causes. Plasma sodium levels were measured before and 1, 3, 5, 15 and 30 days after starting Tolvaptan and then at 3-month intervals. Anamnestic and clinical data were collected.

**Results:**

Mean time spanned 27.3 ± 29.8 months (range 6 months-7 years). Mean plasma sodium levels were within normal range 1, 3 and 6 months after starting Tolvaptan as well as after 1, 2, 3, 5 and 7 years of therapy. Neither osmotic demyelination syndrome nor overcorrection were observed. Plasma sodium levels normalization was associated with beneficial clinical effects. Neurological patients obtained seizures disappearance, improvement in neurological picture and good recovery from rehabilitation. Neoplastic patients were able to start chemotherapy and improved their general condition. Patients did not show hypernatremia during long-term follow-up and reported mild thirst and pollakiuria.

**Conclusions:**

The present study shows that long-term low-dose Tolvaptan is safe and effective in SIADH treatment. No cases of overcorrection were documented and mild side effects were reported.

## Introduction

Hyponatremia is the commonest electrolyte abnormality in hospitalized and community patients and it has been associated with high morbidity and mortality [1, 2]. The syndrome of inappropriate antidiuretic hormone secretion (SIADH) is a major cause of hyponatremia, secondary to a variety of neurological, pulmonary, infective, and neoplastic disorders, as well as drug administration. SIADH may be associated with symptoms such as nausea, vomiting, unstable gait, vertigo, disorientation, and impairment of cognitive functions but it can also present with severe neurological signs, such as seizures and loss of consciousness [1–3].

Conventional SIADH treatment associated with mild to moderate hyponatremia consists of fluid restriction and rarely demeclocycline or urea, whereas hypertonic saline is employed in cases of severe and symptomatic hyponatremia [1, 2]. Tolvaptan is a selective antagonist of the vasopressin V2-receptor that blocks vasopressin activity on renal collecting tubules resulting in an increase in plasma sodium concentrations [1, 2]. The drug is approved for the treatment of adult patients with SIADH, but its use is limited. Tolvaptan starting dose is usually 15 mg/day, but recent clinical experience evidenced that lower doses may be sufficient to normalize sodium plasma levels and suggested a starting dose of 7.5 mg/day [4–6]. At present, only few reports in literature describe chronic SIADH long-term treatment with low-dose Tolvaptan [7–9]. We therefore aimed at better characterizing safety and efficacy of long-term low-dose (<15 mg/die) Tolvaptan treatment in 11 patients with chronic hyponatremia SIADH-related and reviewed related literature.

## Material and methods

We retrospectively evaluated patients who received chronically low-dose Tolvaptan (Samsca, Zhejiang Otsuka Pharmaceutical, Tokyo, Japan) therapy for chronic hyponatremia secondary to SIADH (Tab. 1). The inclusion criteria were:

- SIADH diagnosis with plasma sodium levels <135 mmol/L for ≥30 days;
- treatment with Tolvaptan for >3 months at daily doses <15 mg/day (defined as low-dose Tolvaptan).

Exclusion criteria were: age <18 years old; medication interval >1 week; severe liver or kidney dysfunction before treatment.

SIADH was diagnosed according to the guideline criteria [10]. All patients had been unsuccessfully previously treated with conventional treatments: i.v. and/or enteral sodium supplementation and water restriction (<1.5 l/d) before Tolvaptan administration. Fluid intake was not limited and no extra oral sodium supplements were indicated. In patients receiving enteral nutrition, water daily balance was carefully monitored. All patients routinely took other types of drugs including those favouring hyponatremia (i.e., anti-epileptics). Because some SIADH causes may be transient in nature (i.e., subarachnoid hemorrhage) all patients received dosage adjustment according to plasma sodium levels in the short and long-term to see if the hyponatremia recurred as evidence of chronic SIADH.

Plasma sodium levels were measured before starting Tolvaptan and then at 1, 3, 5, 15 and 30 days. Natremia was then measured at 3-month intervals. Anamnestic and clinical data were collected before and during treatment: age, sex, SIADH cause, blood pressure, heart rate, body weight and symptoms due to hyponatremia. In addition, the following laboratory data were collected: glucose, potassium, creatinine, serum urea nitrogen, uric acid, alanine aminotransferase.

In patients evaluated during traumatic brain injury (TBI) post-acute phase, clinical picture during Tolvaptan treatment was measured by the following scales: Functional Independence Measure (FIM) scoring from 18 (complete dependence) to 126 (complete independence); Disability Rating Scale (DRS) scoring from 0 (no disability) to 9 (extreme vegetative state); Levels of Cognitive Functioning (LCF) with 8 cognitive levels assessing the patient’s cognitive function (8 = purposeful, appropriate response) [11]. Modifications in performance status of oncologic patients during Tolvaptan therapy was assessed by the ECOG Scale (Eastern Cooperative Oncology Group) [12]. Sodium overcorrection was defined by a Na^+^ increase >10 mmol/L during the first 24 h and >8 mmol/L during every 24 h thereafter [8]. Hyponatremia was divided into: mild (Na^+^ between 130 and 135 mmol/L), moderate (Na^+^ between 125 and 129 mmol/L) and severe (Na^+^ <125 mmol/L) [10].

Statistical analysis was performed by means of the Fisher’s exact test for qualitative measures, by *T* test for parametric distribution of quantitative measures and by Kruskal–Wallis test for non-parametric distribution of quantitative measures. *P* values <0.05 were considered to indicate statistical significance. The study was approved by the local ethical committee and informed consent was obtained from the subjects or next of kin.

The drug was administered orally in 7 cases and enterally by percutaneous endoscopic gastrostomy (PEG) in 4 cases.

## Results

We evaluated 11 patients (4 F, 7 M, mean age = 55.72 ± 22.8 years). SIADH was due to neurological causes in eight individuals (4 severe TBI; 1 subarachnoid hemorrhage; 1 cerebral hemorrhage; 1 stereotactic radiosurgery for glioma recurrence; 1 multi-infarct encephalopathy), to neoplastic causes in 2 (small cell lung cancer, SCLC), and to idiopathic cause in one patient (Table 1). Patients started oral Tolvaptan at 15 mg/d in three cases (2 post-acute TBI patients and 1 neoplastic subject), 7.5 mg/d in seven cases, and 3.75 mg/d in one neurological case.

**Table 1:** Characteristics of SIADH patients

Pts, sex	Age (yr)/ BMI (kg/m^2^)	SIADH Causes	Antiepileptic drugs	Other drugs	Sodium at Tolvaptan start (mmol/L)	Tolvaptan dose		
						Initial	At 15 days	At last follow-up
1, M	64/26.4	Severe TBI	Carbamazepine	Amlodipine, Ramipril, Baclofene	124	15 mg/d	7.5 mg/d	7.5 mg/d
2, F	35/21.88	Severe TBI	Lamotrigine, Levetiracetam	Cortisone acetate L-tiroxin, Propranolol, L-dopa, Benserazide, Baclofen	124	15 mg/d	15 mg/d, 7.5 mg/d at 2 mo	7.5 mg/d for 3d/wk and 3.75 mg/d for 4d/wk
3, M	28/ 22.28	Severe TBI	/	/	130	7.5 mg/d	7.5 mg/d	7.5 mg/d at 6 mo, then STOP
4, M	28/ 25.18	Previous severe TBI and politrauma (23 yr old)	Lamotrigine, Levetiracetam, Lacosamide	Atorvastatin, Bisoprolol, Warfarin, Vitamin D	124	7.5 mg/d	7.5 mg for 4 days a wk (30 mg/wk)	7.5 mg/d
5, F	52/ 18.46	Intraparenchymal cerebral hemorrhage	Lamotrigine, Levetiracetam	Vitamin D, Ramipril, Paroxetine	126	7.5 mg/d	7.5 mg/d	7.5 mg/d for 2/wk and 15 mg/d for 5d/wk
6, M	85/ 22.72	Subarachnoid hemorrhage consequent to TBI	Lamotrigine	Warfarin, Irbesartan, Amlodipine, Tiotropium, Trazodone, Tamsulosine	132	7.5 mg/d	3.75 mg/d	3.75 mg/d
7, M	84/ 27.35	Multi-infarct Encephalopathy	/	Aspirin, Amlodipine, Metoprololol, Canrenone, Gabapentin, Tamsulosine	129	7.5 mg/d	7.5 mg/d	7.5 mg/d
8, M	27/ 22.83	Radiosurgery in previous surgical removal of a brainstem astrocytoma (1995, and 2001).	Lamotrigine	Idrocortisone Vitamin D	131	3.75 mg/d	3.75 mg/d	3.75 mg/d
9, F	77/ 25.5	Lung microcytoma with hepatic and bone metastases (stage IV)	/	Amlodipine, Clopidogrel, Esomeprazole, Chemotherapy (Carboplatin-Etoposide)	126	15 mg/d	15 mg/d	7.5 mg/d at 3 mo, than, then gradual reduction until STOP at 6 months
10, M	64/ 25.8	Locally advanced lung microcitoma	/	Amlodipine, Ramipril, Oxycodon, Carboplatin- Etoposide	132	7.5 mg/d	7.5 mg/d	7.5 mg/d
11, F	69/ 24.6	Idiopathic	/	Bisoprolol, Vitamin D, Yearly zolendronate, Pantoprazole	129	7.5 mg/d	7.5 mg/d	7.5 mg/d

*F* Female; *M* Male; *TBI* Traumatic brain injury; *pts* patients; *yr* years old; *mo* months; *wk* week; *BMI* Body Mass Index; *SIADH* syndrome of inappropriate antidiuretic hormone secretion

Six out of eight neurological SIADH patients had seizures treated with antiepileptic drugs. In these patients, hyponatremia was associated with seizure crisis (6 cases), cognitive impairment and poor motor initiative (7 cases), drowsiness (7 cases) and reduced response to the rehabilitation (3 cases).

In neoplastic patients, hyponatremia delayed the start of chemotherapy treatment, that was possible after Tolvaptan administration; in one neoplastic patient hyponatremia caused a single seizure episode with fall and left posterior glenohumeral dislocation. In idiopathic SIADH, hyponatremia caused losses of consciousness and repeated falls with bone fractures requiring some hospitalizations.

Mean plasma sodium levels before starting Tolvaptan were 127.9 ± 3.2 mmol/L (range: 124–132 mmol/L). In particular, three patients had severe, 4 moderate and 4 mild hyponatremia (130 mmol/L ≤ Na^+^ <132 mmol/L) (Table 1). Basal sodium plasma levels were slightly, but not significantly, lower in the three patients starting with Tolvaptan 15 mg/d (mean = 124.7 ± 1.15 mmol/L) compared to the eight patients starting with Tolvaptan 7.5 or 3.75 mg/d (mean = 129.1 ± 2.9 mmol/L). Mean plasma sodium levels significantly increased to 132.6 ± 3.7 mmol/L (*p* < 0.01 vs. basal levels) (range: 128–139 mmol/L) and to 135.1 ± 2.7 mmol/L (*p* < 0.01 vs. basal levels) (range: 131–139 mmol/L) after 1 and 3 days of Tolvaptan treatment, respectively.

The sodium correction rate was slightly but not significantly higher in the 15 mg/d as compared to 7.5 mg/d group after 1 day group (mean: 6.7 ± 1.2 mmol/L and 4.0 ± 2.0 mmol/L, respectively). Similar results were found after 3 days (mean: 9.7 ± 4.0 mmol/L and 6.3 ± 1.8 mmol/L).

The sodium correction rate after 1 day in patients with severe hyponatremia was slightly but not significantly higher as compared to patients with mild-moderate hyponatremia (mean: 5.3 ± 1.2 mmol/L and 4.9 ± 2.5 mmol/L, respectively). Similar results were found after 3 days (mean: 11 ± 1.7 mmol/L and 5.8 ± 1.5 mmol/L, respectively). All three patients starting with 15 mg/d dose had sodium plasma levels ≥130 mmol/L after 1 day of treatment vs. 6 out 8 individuals on 7.5/3.75 mg/d dose (however 4 out 8 patients had Na^+^ basal levels between 130 and 132 mmol/l and the difference was not statistically significant). After 3 days of treatment, all individuals presented with plasma sodium levels ≥130 mmol/L (Table 2).

**Table 2:** Median sodium plasma levels and number of patients reaching normonatraemia during Tolvaptan treatment

Days or Months	*N°* of patients	Plasma sodium levels: Median (IQR)	Patients with plasma sodium levels ≥ 135 mmol/L: *N°* (%)	Patients with plasma sodium levels ≥ 134 mmol/L: *N*° (%)	Patients with plasma sodium levels ≥ 130 mmol/L: *N*° (%)
0 day	11	129 (5.5)	0	0	4 (36.4%)
1 day	11	132 (4.5)	3 (27.2%)	4 (36.4%)	9 (81.8%)
3 days	11	136 (4)	6 (54.5%)	7 (63.6%)	11 (100%)
5 days	11	137 (6)	7 (63.6%)	8 (72.7%)	11 (100%)
15 days	11	139 (5)	9 (81.8%)^b,c^	9 (81.8%)^b,c^	11 (100%)
30 days	11	137 (3)	10 (90.9%)^c^	11 (100%)	11 (100%)
3 months	11	139 (5)	9 (81.8%)^b, c^	10 (90.9%)^b^	11 (100%)
6 months	11	139 (2.5)	10 (90.9%)^b^	10 (90.9%)^b^	11 (100%)
12 months	5	138 (6)	4 (80%)^b^	4 (80%)^b^	5 (100%)
18 months	5	138 (2)	4 (80%)^a^	4 (80%)^a^	5 (100%)
24 months	5	136 (2)	5 (100%)	5 (100%)	5 (100%)
30 months	4	136.5 (2.5)	4 (100%)	4 (100%)	4 (100%)
36 months	4	138 (0.75)	4 (100%)	4 (100%)	4 (100%)
48 months	4	137 (4)	4 (100%)	4 (100%)	4 (100%)
60 months	4	138.5 (7.5)	3 (75%)^a^	3 (75%)^a^	4 (100%)
72 months	1	141 (0)	1 (100%)	1 (100%)	1 (100%)
84 months	1	141 (0)	1 (100%)	1 (100%)	1 (100%)

*N°* Number; *IQR* Interquartile Range

^a^the patient reduced Tolvaptan dose by himself/herlsef determining hyponatremia (132 and 133 mmol/L, respectively)

^b^one TBI patient increasing periodically antiepileptic drugs dose to reach anticonvulsant therapeutic range. Therefore Tolvaptan dose was increased gradually during follow-up

^c^the patient with idiopathic SIADH refused to increase Tolvaptan dose because of the presence of thirst and pollakiuria for 1 year. She manteined always the same Tolvaptan dose (7.5 mg/d)

### Follow-up

**Fig. 1:**
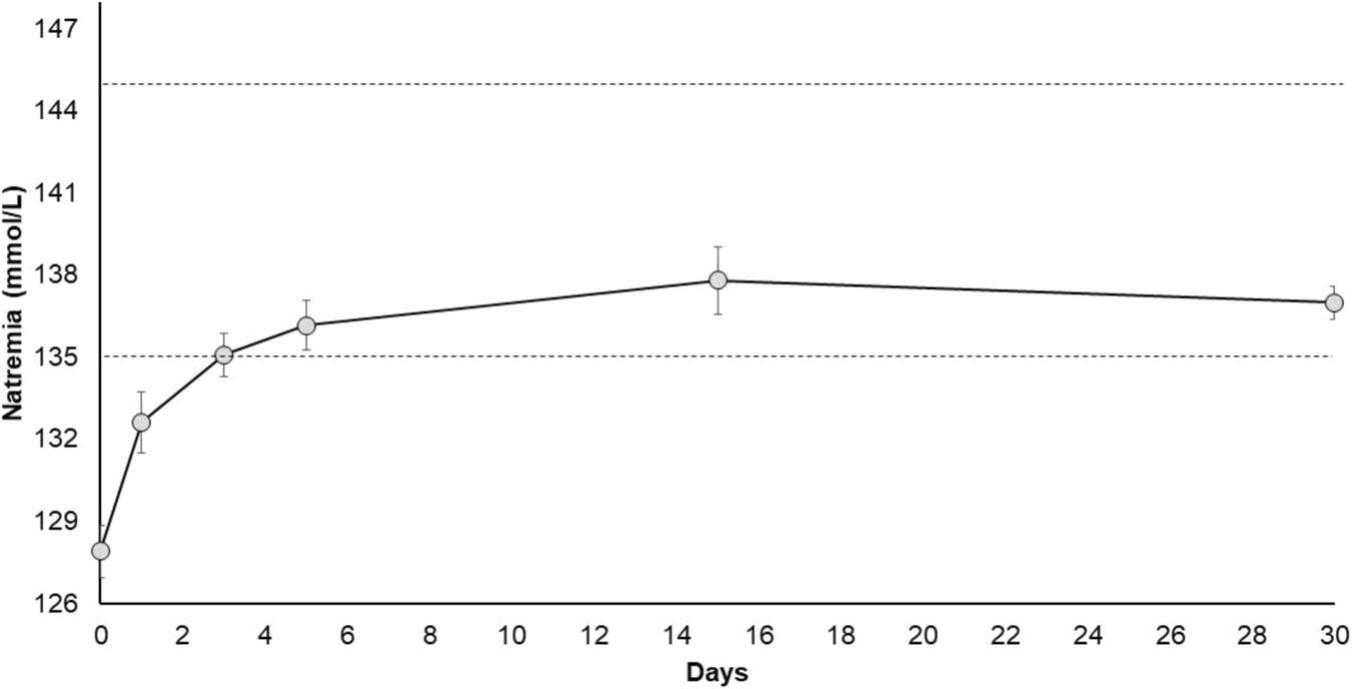
Mean sodium levels over 30 days from Tolvaptan start. Gray dots: mean sodium levels of 11 patients ± standard error of the mean

**Fig. 2:**
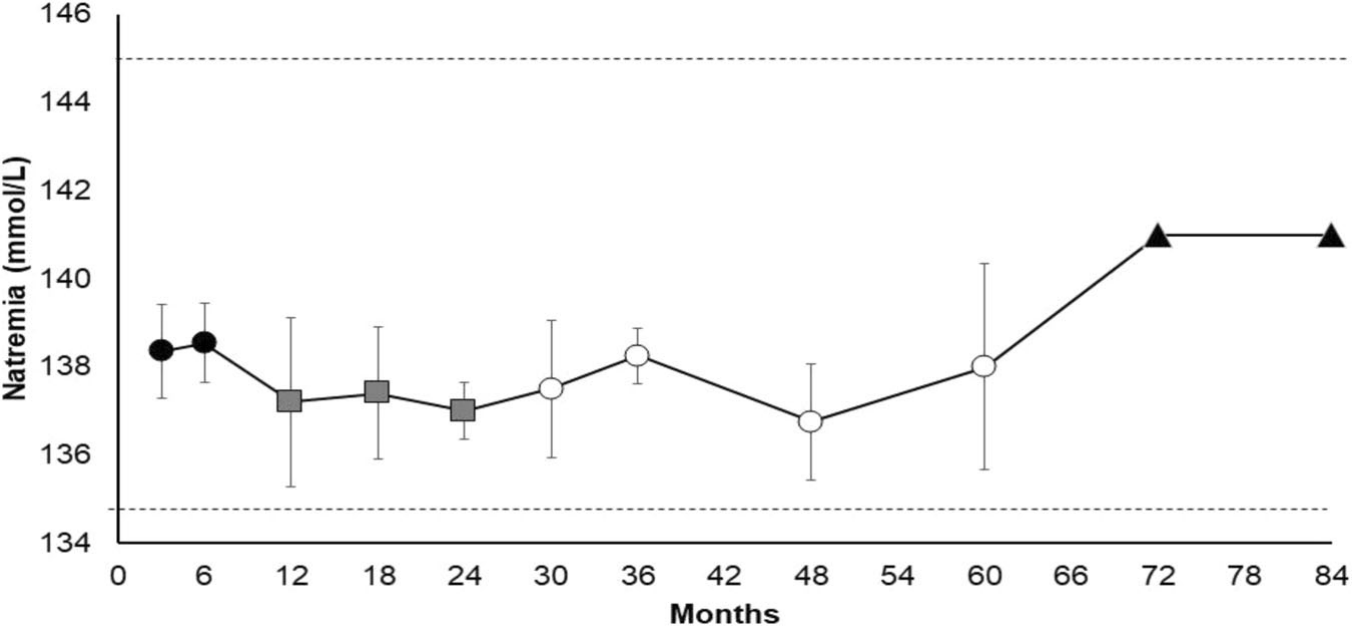
Mean sodium plasma levels under Tolvaptan treatment for more than 30 days. Black dots: mean sodium plasma levels of 11 patients. Gray squares: mean sodium plasma levels of five patients. White dots: mean sodium plasma levels of four patients. Black triangles: sodium plasma levels of one patient. Data are displayed as mean sodium plasma levels ± standard error of the mean

**Fig. 3:**
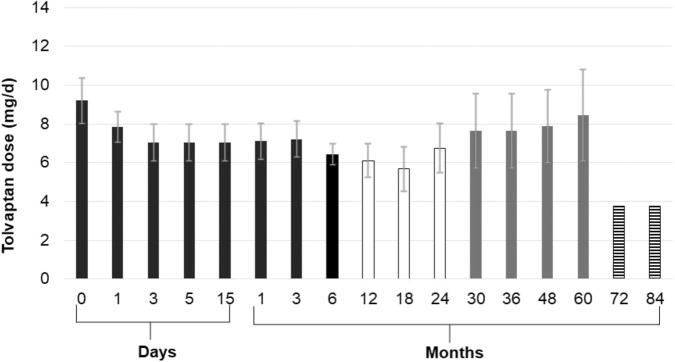
Mean Tolvaptan dose (mg/d) during follow-up. Data are displayed as mean Tolvaptan dose ± standard error of the mean. Black bars: mean Tolvaptan dose of 11 patients. White bars: mean Tolvaptan dose of five patients. Gray bars: mean Tolvaptan dose of four patient. Striped bars: mean Tolvaptan dose of one patient

We followed all patients up for at least 6 months (from 6 to 84 months) with a mean treatment time of 27.3 ± 29.8 months and sodium plasma levels maintained within a low-normal range (Figs. 1 and 2). Median plasma sodium levels at each point are described in Table 2, as well percentage of patients reaching normonatraemia during follow-up. Two patients managed to stop Tolvaptan and plasma sodium levels remained within reference range after drug withdrawal. In particular, one TBI patient stopped therapy after 6 months thanks to an optimal response recovery after rehabilitation and improvement of neurological conditions, whereas one neoplastic patient stopped Tolvaptan after 6 months thanks to response to chemotherapy. In patients receiving longer treatment, sodium plasma levels were stable up to 7 years of therapy, with few dose adjustments (Figs. 2 and 3), frequently concomitant with modifications in anti-epileptic drug treatment (Table 2).

### Clinical benefits

In all patients, normalization of sodium plasma levels was associated with beneficial clinical effects (Table 3). The eight neurological patients obtained seizures disappearance and improvement in neurological picture. Patients and their families reported an improvement in attention, memory, motor skills and quality of life. Two patients treated in the TBI post-acute phase showed little improvement in cognitive and motor functioning scales, with slight but not significant changes in the evaluated scales (at rehabilitation admission FIM = 18/126, LCF = 3/8, DRS = 22/29; at rehabilitation discharge FIM = 30–35/126, LCF = 3–4/8, DRS = 20–16/29). One post-TBI patient had a good response to rehabilitation, presenting with extremely severe disability at admission (FIM = 18/126; LCF = 3/8, DRS = 21/29) and moderate disability at discharge (FIM = 101/126; LCF = 8/8, DRS = 4/29).

**Table 3:** Clinical benefits after plasma sodium correction with Tolvaptan

Neurological Pts: *N°*, Sex and Age	Seizure disappearance	Rehabilitation scores improvement	Drowsiness reduction	Cognitive and motor skill improvement
1, M, 64 yr	Yes	Yes	Yes	Yes
2, F, 35 yr	Yes	Yes	Yes	Yes
3, M, 28 yr	NA	Yes	Yes	Yes
4, M, 28 yr	Yes	NA	Yes	Yes
5, F, 52 yr	Yes	NA	NA	NA
6, M, 85 yr	Yes	NA	Yes	Yes
7, M, 84 yr	NA	NA	Yes	Yes
8, M, 27 yr	Yes	NA	Yes	Yes

Neoplastic Pts: *N°*, Sex and Age	Possibility of starting chemotherapy	ECOG improvement	Drowsiness/ Seizures disappearance	Cognitive and motor skill improvement
9, F, 77 yr	Yes	Yes	Yes/ Yes	Yes
10, M, 64 yr	Yes	No (onset of chemotherapy side effects that delayed subsequent cycles)	Yes/ NA	Yes

Idiopathic Pt: *N°*, Sex and Age	Loss of consciousness	Falls and fractures disappearance	Drowsiness reduction	Cognitive and motor skill improvement
F, 69 yr	No	Yes	Yes	Yes

*Pts* patients; *Pt* patient; *N°* Number; *F* Female; *M* Male; *yr* years old; *ECOG* Eastern Cooperative Oncology Group; *NA* not applicable because those symptoms/clinical conditions were not present before Tolvaptan treatment

After the start of Tolvaptan treatment, neoplastic patients were able to start chemotherapy and their general condition improved. ECOG improved from 2 to 0 points in one neoplastic patient thanks to chemotherapy and hyponatremia normalization and she obtained seizures disappareance. In this patient Tolvaptan was subsequently discontinued. The other neoplastic patient had a stable ECOG (1 point) but developed neutropenia during chemotherapy that delayed subsequent cycles (Table 3).

The patient with idiopathic SIADH reported improvement in general well-being and physical performance; the losses of consciousness and the repeated falls did not occur any more (Table 3).

### Adverse events

Osmotic demyelination syndrome and overcorrection were not observed during the first 24–72 h nor during long-term follow-up. No changes in body weight, heart rate, blood pressure and biochemical parameters were observed during follow-up. One patient reported thirst and pollakiuria for 1 years. No liver toxicity occurred. After 18 months from the start of Tolvaptant treatment, epileptic crisis developed in one male neurological patient displaying concomitant hyponatremia (132 mmol/L) because of Tolvaptan dose reduction made by himself (from 7.5 mg/d for 4 day a week to 7.5 mg/d for 2 days a week). Sodium plasma levels returned within normal range after the subsequent increase of Tolvaptan dose and seizures disappeared. The same episode occurred in one female neurological patient after 60 months of treatment (Na^+^ 133 mmol/L after Tolvaptan dose reduction made by herself). Seizure disappeared and Na^+^ normalized after increase in Tolvaptan dose.

## Discussion

The present study demonstrates safety and efficacy of short- and long-term treatment (up to 84 months) with low-dose Tolvaptan (3.75–15 mg/d) in patients with chronic SIADH due to neurological, idiopathic and neoplastic causes, not responsive to conventional treatment. Indeed, in our study all patients achieved normal sodium plasma levels despite Tolvaptan was started at doses lower that those recommended by current guidelines. In addition, few adverse events were recorded. As a first-line treatment, the traditional option is fluid restriction. However, often due to specific patient characteristics, this strategy is not possible to apply, or on the other hand, it is not very effective, sometimes because of the low therapeutic adherence. In consequence, we must resource to alternative pharmacological therapies, such as vaptans or urea. In clinical practice urea is not widely extended maybe because of the lack of convincing evidences with limited and heterogeneous studies [13]. Therefore we chose to use tolvaptan, also driven by the fact that urea is not palatable with poor adherence to therapy.

Nowadays, only few data on long-term Tolvaptan administration in SIADH have been reported (Table 4). In addition, only one report described the efficacy of low-dose Tolvaptan in 4 SIADH patients with age >90 years old with a follow-up of 2 years [7–9] whereas the other papers report data on chronic treatment with standard dose of Tolvaptan. To our knowledge, our study reports the longest follow up with low-dose Tolvaptan (up to 84 months) and, differently to other works, in the present study all patients used Tolvaptan dose <15 mg/d at the last follow-up.

**Table 4:** Revision of the literature on long-term Tolvaptan treatment (>12 months) in SIADH

Authors	Patients (*N°*)	Patients’ age (yr)	Tolvaptan starting dose (mg/d)	Tolvaptan up-titration to 30–60 mg/d	Tolvaptan dose at last follow *p* (mg/d)	Tolvaptan dose <15 mg/d over time	Maximal treatment duration (years)	Clinical symptoms improvement
Liu et al. [5]	4 SIADH 2 CHF 1 CRF	96.7 ± 1.6	7.5	Yes^a^	7.5 or 15	Yes	2^a^	NA
Büttner et al. [6]	1 SIADH	80	15	Yes	15	No	6	Disappearance of dizziness/ syncope
Berl et al. [7]	58 with SIADH; 20 with cirrhosis; 33 CHF	64.6 ± 15.0^a^	15	Yes	nn	No	4.1^a^	NA

^a^only SIADH patients; *nn* not reported; *NA* Not Applicable. *CHF* Chronic Heart Failure; *CRF* Chronic Renal Failure; *yr* years old

Usually, Tolvaptan 15 mg/d represents the starting dose that can be increased to 30–60 mg/d according to sodium plasma levels. In our study, sodium plasma levels normalized both with starting Tolvaptan doses of 15 mg/d (three patients) and with lower doses (7.5 mg and 3.75 mg/d). In addition, doses >15 mg/d were not necessary. These data suggest that in SIADH patients a Tolvaptan dose of 7.5 mg/d might be effective in adequately control natremia in the short-term. Our results are in line with those reported by a study evaluating 23 SCLC patients with hyponatremia due to SIADH. In these settings, sodium plasma levels were similar in patients receiving 3.75 mg/d and in those receiving >3.75 mg/d Tolvaptan after 3 days of treatment [14].

Our data suggest that Tolvaptan can be co-administered, even in the long- term, with drugs determining hyponatremia without further adverse events. In particular, in epileptic subjects, keeping normal sodium plasma levels improved clinical status and led to seizures disappearance. These aspects may reduce medical costs due to hospitalization and improve medical condition [13–20]. In neurological patients, natremia normalization significantly improved neurological symptoms and quality of life of the patients and their caregivers. Verbalis et al. indicated that SIADH patients treated with Tolvaptan 15 mg/d (or higher doses) had a statistically significant clinical improvement in the Physical Component Summary (PCS) after 30 days of therapy in comparison with placebo. They also showed that Mental Component Summary (MCS) improvement was higher in Tolvaptan group in comparison with placebo, and the difference was near to statistical significance [21].

Our study supports the hypothesis that Tolvaptan may positively impact the outcome of hyponatraemic neoplastic patients, allowing to start chemotherapy earlier as previously described [22]. In addition, patients treated with chemotherapic drugs often need adequate hydration, contraindicating fluid restriction. In our study, natremia was well controlled by low Tolvaptan dose even in cancer patients. In addition, chemotherapy may induce SIADH (i.e., cisplatin) [23]. Our study also suggests that Tolvaptan reduction should be attempted as soon as a tumor response to chemotherapy is recorded. De Las Peñas et al. suggest that in SCLC patients Tolvaptan dose should be reduced from 15 mg/d to 7.5 mg/d if hyponatremia improves after 7 days of treatment, and then stopped in case of further improvement after 14 days [13]. This approach may reduce the risk of overcorrection and medical costs, improving symptoms, morbidity and mortality at the same time [14–20, 23]. Our data also suggest that Tolvaptan dose reduction is possible when hyponatremia causes can be successfully managed, allowing an improvement in patient clinical picture.

The present data indicate that long-term low-dose Tolvaptan (<15 mg/d) is safe, both in the short- and in the long-term. Indeed, in our series overcorrection and osmotic demyelination syndrome did not occur. Indeed, when the plasma sodium concentration increases too rapidly, osmotic demyelination syndrome may develop and permanent brain damage may occur [14]. However, overcorrection may also depend on the severity of basal hyponatremia [1, 2, 13, 14] and none of our patients had sodium plasma levels <120 mmol/L at study entry. In addition, in keeping with literature data, sodium correction rate was slightly lower in patients receiving low-dose Tolvaptan (3.75–7.5 mg/d) as compared to patients receiving Tolvaptan 15 mg/d [4–6, 24]. On the opposite, Tzoulis et al. showed similar mean daily correction rates in patients with both 15 mg/d and 7.5 mg/d. This finding may be due to patient selection, since Tolvaptan 7.5 mg/d was delivered to patients with severe hyponatremia [25].

Low Tolvaptan dose was safe also in severe neurological patients in whom the probability of overcorrection is high, due to the lack of thirst. Therefore, such patients require a strict clinical and biochemical monitoring especially in the first phase of treatment. In these patients, PEG was a useful tool for Tolvaptan administration, helping drug management and fluid administration.

Tolvaptan was safe in all treated patients, regardless of age and drug co-administration, and it did not cause any significant change in other biochemical parameters or blood pressure, as previously reported [7, 26]. In particular, long term combination with antiepileptic, anti-hypertensive, and/or antiplatelet/anticoagulant drugs did not change the efficacy neither increased the incidence of adverse effects of these drugs. Indeed, we did not observe serious adverse events in neither the short- nor the long-term period. The most common adverse events were thirst and pollakiuria, both of which disappeared, as previously reported [9, 27]; no liver toxicity occurred.

It is well known that chronic hyponatremia is associated with significant morbidity including increased prolonged length of stay, and increased readmissions to hospital, as well as cognitive dysfunction, gait instability, and fractures [28]. The clinical benefit demonstrated in our patients suggest that treatment of chronic hyponatremia with low dose of Tovalptan may safe and efficacy in preventing morbidity due this conditions. Limits of the study are the small sample size and the retrospective design. Prospective studies with larger numbers of patients are needed to confirm these data

## Conclusions

The present study demonstrates safety and efficacy of short- and long-term treatment (up to 84 months) with low-dose tolvaptan (<15 mg/d) in patients with SIADH due to neurological, idiopathic and neoplastic causes. No cases of overcorrection were found and the most common adverse events were thirst and pollakiuria. Low-dose Tolvaptan can be co-administered with drugs determining hyponatremia without adverse events in both short- and long-term period. PEG was a useful tool for the administration of Tolvaptan in severe neurological subjects.

## Data Availability

Data that support the findings of this study are available on request from the corresponding author.
